# Mucocutaneous Manifestations of Inflammatory Bowel Disease

**DOI:** 10.7759/cureus.17191

**Published:** 2021-08-15

**Authors:** Jose C Alvarez-Payares, Sara Ramírez-Urrea, Laura Correa-Parra, Daniela Salazar-Uribe, Mateo Velásquez-López

**Affiliations:** 1 Internal Medicine, University of Antioquia, Medellin, COL; 2 General Medicine, Fundación Universitaria San Martín, Medellin, COL; 3 Dermatology, Universidad de Antioquia, Medellin, COL; 4 General Medicine, Corporación Para Estudios en la Salud (CES), Medellin, COL

**Keywords:** inflammatory bowel disease, cutaneous crohn’s disease, ulcerative colitis (uc), extraintestinal manifestations in inflammatory bowel disease, cutaneous manifestations of inflammatory bowel disease

## Abstract

Inflammatory bowel disease (IBD) is a chronic and incurable disease, of unknown etiology, associated with an unregulated immune response to environmental triggers in a genetically predisposed host. IBD affects mainly the gastrointestinal (GI) tract and includes Crohn's disease (CD) and ulcerative colitis (UC). However, a large percentage of patients may present with extraintestinal manifestations, including mucocutaneous ones (which are the most common) and dermatologic findings, such as erythema nodosum, pyoderma gangrenosum, and aphthous stomatitis (which are the most frequently occurring). According to pathophysiologic mechanisms, mucocutaneous manifestations of IBD are classified into five categories, namely, specific manifestations, associated manifestations, reactive manifestations, adverse effects of IBD therapy, and malabsorption manifestations. Recognizing such manifestations should not be performed only by a dermatologist but also other specialties such as internal medicine, gastroenterology, among others. This is because these manifestations can present before the IBD diagnosis, even in the absence of GI symptoms. Therefore, these skin lesions could be a fundamental tool for the earlier diagnosis of IBD. This review provides a comprehensive overview of the most common cutaneous manifestations of IBD with a focus on their epidemiology, diagnostic criteria, clinical presentation, and available medical treatment.

## Introduction and background

Inflammatory bowel disease (IBD) is a chronic and incurable disease, of unknown etiology, believed to be associated with an unregulated immune response to environmental triggers in a genetically predisposed host [1]. The prevalence of IBD increased significantly during the second half of the 20th century and the beginning of the 21st century worldwide, primarily in developing countries and the pediatric population [2]. The highest prevalence of IBD was reported in Europe, of 505 cases per 100.000 persons. Since 1990, the incidence of IBD in western countries has stabilized or even started to decrease, but the incidence rate in recently industrialized countries in Asia, Africa, and South America has been increasing [1,3].

IBD includes Crohn’s disease (CD) and ulcerative colitis (UC), which have clear clinical, histological, and endoscopic differences (Table 1). IBD affects mainly the gastrointestinal (GI) tract, however, up to 40% of patients may present with extraintestinal manifestations, which are more common in CD [4], and up to 36.6% have more than one extraintestinal manifestation [5]. In CD, independent predictive factors for extraintestinal manifestations have been identified; these include female, age, and disease activity [5]. In UC, extensive colitis has been significantly associated with extraintestinal manifestations [5]. Recent studies have revealed that extraintestinal manifestations appear more commonly after IBD diagnosis, with a mean time of 92 months [5]. However, 25.8% occur before diagnosis, with a mean time of five months, although in some patients, a difference of up to two years can be observed before the diagnosis of IBD [5]. There is no clear underlying mechanism of extraintestinal manifestations; it has been proposed as a consequence of an intestinal inflammatory process or a genetic disorder that leads to a dysfunctional immunologic response to environmental stimuli [5].

**Table 1:** Comparison of characteristics between UC and CD Adapted from [1] UC: ulcerative colitis; CD: Crohn's disease

	Ulcerative colitis	Crohn’s disease
Distribution	Limited to colon, continuous	From mouth to anus, discontinuous
Rectum	Affected	Frequently spared
Inflammation	Limited to mucous	Transmural
Mucosa	Granular, erythematous, friable, mucous exudates, spontaneous hemorrhages	Small linear or serpiginous, over normal mucosa, cobblestone, fissures
Abdominal pain	Common	Common
Rectal bleeding	Common	Occasional
Diarrhea	Common	Common
Weight loss	Infrequent	Common
Colon cancer risk	Discretely elevated	Lower than colitis
Perianal involvement	Infrequent	Common
Stenosis	Infrequent	Common
Abscesses	Infrequent	Common
Fistulae	Infrequent	Common

## Review

The most common extraintestinal manifestations are mucocutaneous ones, present in up to 22%-75% of patients with CD and 5%-11% of UC patients [6], followed by musculoskeletal ones, eye disease, hepatobiliary disease, and endocrine disease [7]. The most common mucocutaneous manifestations are erythema nodosum, pyoderma gangrenosum, and aphthous stomatitis [8]. According to pathologic mechanisms, IBD’s mucocutaneous manifestations are classified into five categories (Table 2). In this review, we focus on specific mucocutaneous manifestations, reactive manifestations, and IBD-associated manifestations.

**Table 2:** IBD cutaneous manifestations Adapted from [8] IBD: inflammatory bowel disease

Specific manifestations with the same histologic characteristics as the underlying IBD	Continuous Crohn’s disease: perianal and oral
	Metastatic Crohn’s disease
IBD associated manifestations	Aphthous stomatitis
	Erythema nodosum
	Psoriasis
	Acquired epidermolysis bullosa
Reactive cutaneous manifestations that share one or more pathophysiologic mechanisms with IBD, although without the same histologic characteristics as the GI lesions	Pyoderma gangrenosum
	Sweet’s syndrome
	Bowel-associated dermatosis-arthritis syndrome (BADAS)
	Pyodermatitis-pyostomatitis vegetans (PDPSV)
	Synovitis, acne, pustulosis, hyperostosis, osteitis syndrome (SAPHO)
	Pyogenic arthritis, pyoderma gangrenosum, and acne syndrome (PAPA)
Adverse effects of IBD therapy	Adverse reactions: local reactions, perfusion reactions, paradoxical reactions, eczematous and psoriasiform reactions, potentially lethal disorders
	Cutaneous infections: bacterial (erysipelas, cellulitis, abscess), virus (herpes, cytomegalovirus, papillomavirus), fungi, opportunistic infections
	Cutaneous neoplasms: Non-melanoma skin cancer (basal cell carcinoma, squamous cell carcinoma); Cutaneous lymphoma (mycosis fungoides, Sézary syndrome)
Malabsorption manifestations	Stomatitis, glossitis, angular cheilitis, pellagra scurvy, purpura, enteropathic acrodermatitis, phrynoderma, seborrheic dermatitis, nail and hair abnormalities

Specific mucocutaneous manifestations

Specific mucocutaneous manifestations represent an extension of the GI inflammatory process, sharing the same histologic findings [5], including non-caseating granulomas, dermic infiltrates with giant multinucleated cells, lymphocytes, and eosinophils [4]. These are found only in CD, as UC does not extend to external mucosa [8].

Continuous Crohn’s Disease

Crohn’s disease exhibits as an extension of IBD to sites adjacent to the GI tract. It consists of oral and perianal manifestations, with perianal ones usually being the first manifestation of CD [9]. These include abscesses, fistulae, fissures, and ulcers, which considerably contribute to CD morbidity [10].

Perianal lesions are observed in 36% of patients [4,11]. It may present as erythema, fissure, perianal stenosis, fecal incontinence, abscess, and fistulae (these two being more frequent); such lesions may also be observed in a peristomal region and abdominal scars due to laparotomy or umbilical ones creating enterocutaneous fistulae [4,8]. Specific oral lesions are found in 8%-9% of patients; these present primarily as angular cheilitis, linear and deep lip ulcers, lip and tongue fissures, gingival nodules, cobblestone-like oral mucosa, and painful gingivitis [4,11].

Metastatic Crohn’s Disease

It's considered an extension of CD to sites that are not adjacent to the GI tract. These are located mainly in extremities and intertriginous areas and rarely in genitalia and face; however, any area may be involved [11]. It’s characterized by the presence of specific non-caseating granulomatous skin lesions. Clinically, plaques, nodules, ulcers, abscesses, and fistulae may be observed [11]. Major differential diagnoses are erysipelas, cellulitis, and hidradenitis suppurativa [8]. The severity of metastatic lesions is not related to the degree of inflammation of intestinal disease [11] and responds slower to treatment than GI tract lesions [9].

Reactive cutaneous manifestations

Reactive cutaneous manifestations share one or more pathophysiologic mechanisms with IBD, although these do not portray the same histologic characteristics of GI lesions. A theory is a cross-reaction antigenicity between the skin and intestinal mucosa [11]. These lesions are present in both CD and UC [8] and include pyoderma gangrenosum (PG), pyodermatitis-pyostomatitis vegetans (PDPSV), Sweet's syndrome, bowel-associated dermatosis-arthritis syndrome (BADAS). Less frequently, the synovitis, acne, pustulosis, hyperostosis, osteitis syndrome (SAPHO) and the pyogenic arthritis, pyoderma gangrenosum, and acne syndrome (PAPA) are included [8].

Pyoderma Gangrenosum

This occurs in approximately 1%-2% of patients and is more frequently associated with UC. To a greater extent, it affects female patients, Afro-descendant patients, and those with a family history of UC [8]. The pathophysiology isn't completely understood; it's considered to be an autoinflammatory process associated with an innate and adaptative immunity dysfunction. It is characterized, along with Sweet's syndrome as neutrophilic dermatoses due to neutrophil skin accumulation and activation and is less frequently seen in internal organs [8]. Clinical variants that are currently recognized are ulcerative, bullous, pustular, vegetative, and drug-induced. Initially, it presents as papules, pustules, or nodules that rapidly ulcerate, developing a painful lesion with violaceous borders that spread peripherally [8]. Although any body part may be affected, these lesions appear more commonly on legs and peristomal regions usually preceded by trauma, which is called the Patergia phenomenon [8]. This condition is difficult to diagnose due to a large number of differential diagnoses; therefore, a thorough physical examination and biopsy are needed [12]. The biopsy typically shows a neutrophilic infiltrate with peripheral lymphocyte accumulation [8]. Treatment starts with topical steroids, wound care, and calcineurin inhibitors; however, an early start of systemic steroids and cyclosporin may be required [13]. Surgery is reserved for severe or refractory cases [13].

Sweet’s Syndrome

It occurs more frequently in women, in the third to fifth decades of life and is more prevalent in CD (8). While it is mainly characterized by erythematous papules and plaques, during the natural course of the disease, vesicles and pustules in the face, neck, and upper limbs may be observed; rarely, these have also been observed in the esophagus, duodenum, and rectum [8]. In most patients, skin lesions are associated with systemic symptoms, such as fever, arthralgias, myalgias, headache, conjunctivitis, and oral ulcers [8]. Histologically, it is characterized by a diffuse infiltrate with mature neutrophil predominance, typically located in the superior dermis, which rapidly improves after treatment starts [14], or it could spontaneously remit in weeks-months with no scarring [8].

Bowel-Associated Dermatosis-Arthritis Syndrome (BADAS)

It is considered a rare neutrophilic dermatosis, which has been primarily described after laparoscopic jejunoileal bypass surgery for obesity, also as a postoperative complication due to multiple intestinal surgeries in patients with diverticulitis, appendicitis, and IBD patients [4]. The pathogenesis is yet unclear. It has been suggested that it may be due to excessive intestinal bacteria overgrowth that leads to immune complex deposits in the skin and synovial membrane, inducing inflammation [8]. Clinically, it’s characterized by recurrent episodes of asymmetric, non-destructive arthritis, usually of polyarticular nature on upper limbs, which may be associated with tenosynovitis [15]. Skin lesions are generally distributed across the upper torso and upper limbs, characterized by painful, erythematous papules and plaques, occasionally pustules, and aseptic vesicles. These are associated with fever, malaise, abdominal pain, diarrhea, and malabsorption [4,15-16]. Histologically, they present as perivascular neutrophil infiltrates, with histiocytes that contain polymorphonuclear fragments known as nuclear dust in its cytoplasm and dermic edema [4,17]. Treatment includes antibiotics, systemic steroids, dapsone, and sulfapyridine, and reconstitution of normal intestinal anatomy through surgery [15-16].

Pyodermatitis-Pyostomatitis Vegetans (PDPSV)

It’s a rare IBD mucocutaneous sign that occurs in a 3:1 relationship (male:female) [4]. It's considered a specific IBD marker, associated with UC in 53% and CD in 11% of cases; however, it has been associated with leukemia, alcohol use disorder, diffuse T cell lymphoma, chronic malnutrition, HIV infection, and even in healthy subjects [18]. Predisposing factors include bacterial infections, halogens, tattoos, and foreign body reactions in patients with immune system disorders [19]. It may present with either mucose involvement or isolated cutaneous involvement, or both [4]. Clinically, it's characterized by multiple friable pustules, which are usually small and painless, and exophytic plaques over an erythematous base, which tend to evolve to superficial ulcers, which may be painful, known as snail trails, in the oral cavity [19]. Cutaneous lesions present as papules, pustules, vesicles, and crusts, which coalesce onto vegetant plaques with raised borders, which mainly affect the face, scalp, axillae, groin, and, less frequently, the abdomen, thorax, and distal zone of limbs [4,19]. Histologically, it presents as pseudoepitheliomatous hyperplasia, dermic and epidermic neutrophils, with multiple eosinophilic microabscesses, usually without granulomas [20]. In up to 90% of cases, it's associated with peripheral eosinophilia; hence; it can be used for diagnostic purposes [20]. Direct and indirect immunofluorescence are usually negative [20-21]. Definitive treatment is based on the management of the underlying disease; antibiotic treatment is only recommended in culture-based cases [19].

Synovitis, Acne, Pustulosis, Hyperostosis, Osteitis Syndrome (SAPHO)

It presents primarily in young patients. It’s a seronegative spondyloarthritis [8], characterized by: a) Synovitis, which is frequently non-erosive inflammatory arthritis [22]; b) Acne conglobate or fulminans, which may also include manifestations such as dissecting cellulitis of the scalp and hidradenitis suppurativa [8]; c) Osteitis, with bone pain and edema due to cortex/medullary inflammation or both; d) Hyperostosis, with bone overgrowth due to endosteum and/or periosteum proliferation is a result of trabeculae and cortical thickening with medullary canal narrowing. Osteolytic lesions may coexist when hyperostosis and osteitis are present [22]. Osteoarticular involvement is insidious, with pain, morning stiffness, edema, persistent fever, and acute phase reactants elevation [22]. Non-steroidal anti-inflammatory drugs (NSAIDs) are frequently used during the diagnostic phase and are the first line for pain relief [22]. Intra-articular glucocorticoids and systemic glucocorticoids have a transitory efficacy, with the relapsing disease when dose reduction or withdrawal is attempted [22]. Antimicrobial treatment, such as doxycycline, is an alternative due to the theoretical role of Propionibacterium (P.) acnes in disease pathophysiology [22]. Some immunosuppressants have had controversial results, and bisphosphonates have shown efficacy for complete and sustained remission for osteoarticular involvement, with no efficacy on cutaneous manifestations [22]. Recent studies have shown that Tumor necrosis factor-alpha (TNF-α) inhibitors are effective for complete remission of bone, articular, and cutaneous involvement, as it is a potent regulator of cytokines such as IL-1, IL-6, IL-8, which are altered in SAPHO patients [22].

Pyogenic Arthritis, Pyoderma Gangrenosum, and Acne Syndrome (PAPA)

It’s a dominant autosomal autoinflammatory syndrome due to mutations in proline-serine-threonine phosphatase-interacting protein 1 (PSTPIP1) [23], first reported in 1997. It's characterized by aseptic inflammation of the skin and joints, mainly in the elbows, knees, and ankles. Articular involvement presents as a painful, recurrent, sterile monoarthritis triggered by trauma, although it can also present spontaneously [24]. Joint erosions may be observed, which could lead to joint destruction [24]. These generally present in childhood as the first sign of the disease and tend to improve in adulthood [24]. As previously mentioned, cutaneous involvement presents as pyoderma gangrenosum and acne. Acne presents primarily in puberty, with variable severity, usually severe nodular/cystic acne, which usually leaves scars when no treatment is given [24]. The Pathergy sign is usually positive [4,24]. Systemic corticosteroids and different immunosuppressants, such as IL-1 antagonists and TNF α inhibitors, are some of the available resources for this entity [23].

Associated cutaneous manifestations

Associated cutaneous manifestations are those that are relatively frequent in patients with IBD. Pathophysiologic mechanisms are related to the chronic inflammatory state and the expression of certain genes of the human leukocyte antigen (HLA) such as HLA-DR2 and HLA-B27 [4,8].

Aphthous Stomatitis

It is present in approximately 10% of patients with IBD, most frequently in CD, but the recurrence is more frequent in UC [4,25]. Oral ulcers may be a consequence of CD extension, with classical granulomatous inflammation, or secondary to nutritional deficiency due to malabsorption secondary to IBD [26]. A recent study found that B12, folic acid, and iron deficiency, whether they occur simultaneously or alone, are associated with aphthous stomatitis in patients of all ages [26]. It’s characterized by multiple painful and round/oval ulcers, with a yellow pseudomembranous base and erythematous borders [27]. These ulcers are usually located in oral or lip mucosa; its typical aspect makes histopathological evaluation unnecessary in patients with IBD. Nonetheless, border biopsy and culture may be useful in persistent/recurrent/refractory lesions and/or patients without a clear IBD diagnosis [8,27].

Erythema Nodosum

It’s the most common cutaneous manifestation, presenting in up to 3%-10% of patients with UC and 4%-15% of CD patients, occurring most frequently in female patients aged between 25 and 40 years [28]. It’s considered a delayed cellular hypersensitivity reaction, triggered by diverse antigenic stimuli [8], which starts with an abrupt eruption of erythematous, warm, painful, non-ulcerative nodules that occasionally coalesce, creating erythematous plaques of approximately 1-5 cm [8]. Its distribution is usually bilateral and symmetrical, and the color usually changes - initially as a bright red, then purpuric, and yellow/violaceous thereafter. It may present with systemic symptoms such as fever, myalgias, arthralgias, headache, GI symptoms, fatigue, or cough, and other less common manifestations such as hepatomegaly, splenomegaly, and pleuritis [8]. These nodules are usually located on the extensor surfaces of lower limbs; however, any area of the body may be affected - face, torso, and upper limbs [8]. Its diagnosis is usually based on clinical presentation alone, with no need for biopsy [8]. The nodules usually persist for three to six weeks, disappearing without any scarring. During the course of the disease, leg elevation, and rest are recommended, and in case of any pain, NSAIDs are the first line of treatment [8,29].

Psoriasis

Seven to 11% of patients with IBD develop psoriasis, an erythematous/scaly disease that occurs more frequently in CD than in UC [8]. Patients with psoriasis have a higher predisposition to develop IBD; likewise, there’s a high risk of iatrogenic psoriasis lesions in patients with IBD undergoing anti-TNF treatment [8]. Plaque psoriasis is the most common subtype (vulgar psoriasis or chronic plaque psoriasis [30]. Lesions are usually monomorphic, with well-defined erythematous plaques covered by silver scales that may be scarce or present as erythroderma affecting the whole body surface [30]. It may affect any skin area but typically locates in extensor surfaces of the forearm and legs, periumbilical, perianal, retroauricular areas, and scalp [30]. Anti-TNF has been successfully used for both psoriasis and IBD management, as TNF has an important role in the pathogenesis of both diseases - infliximab, adalimumab, and certolizumab may all induce remission of both diseases [31].

Epidermolysis Bullosa Acquisita (EBA)

It is an autoimmune bullous disease caused by antibodies against type VII collagen [8]. Thirty percent of patients with EBA also present with IBD, which is believed to be secondary to chronic intestinal inflammation, which may result in autoantibody development against type VII intestinal collagen [4]. These autoantibodies react against type VII collagen in the dermo-epidermal junction, inducing the development of bullae, a phenomenon known as epitope spreading [4]. The two most common presentations of EBA are the classic non-inflammatory one and bullous pemphigoid-like EBA. Classical EBA is characterized by skin fragility, prone to superficial trauma with erosions, vesicles, bullae without inflammation, hyperpigmentation, and scarring that mainly occurs on the hand's dorsum, elbows, knees, and feet [32]. Bullous pemphigoid-like EBA presents as vesicle/bullae eruption, with erythema and pruritus. These lesions generally affect the torso, intertriginous areas, and extremities; no frailty or scarring is observed [32].

**Figure 1:**
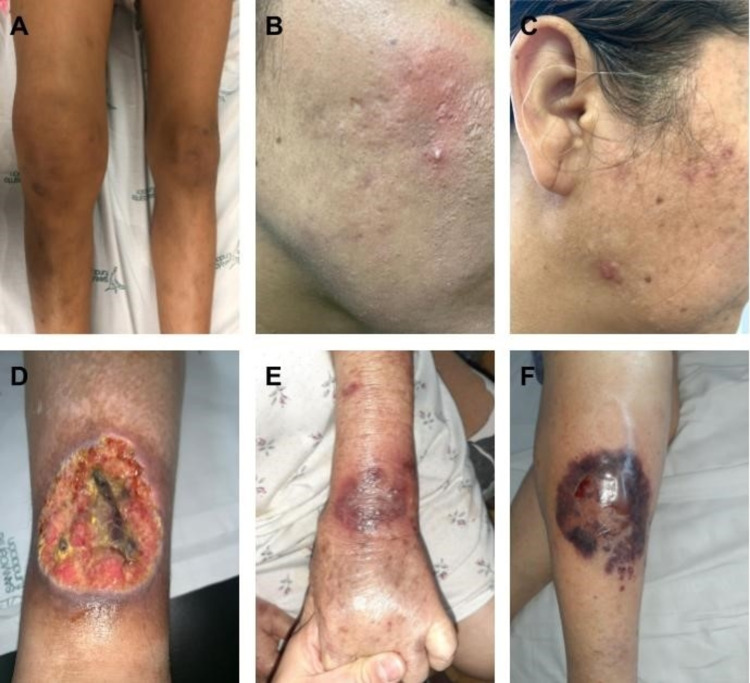
A) Papa syndrome: Pyogenic arthritis. Edema, joint effusion, functional limitation of the knee; B-C) Papa syndrome: Acne. Post-inflammatory hyperpigmentation. Presence of facial erythema, erythematous papules, pustules, and atrophic scars type: boxed and ice pick. D) Papa syndrome: Pyoderma gangrenosum. Ulcer with a raised violet erythematous border, well-defined, with a yellowish background, painful on the front of the leg. E) Acquired epidermolysis bullosa. This clinical presentation is similar to bullous pemphigoid with tense bullae, erosions, and crusts over an erythematous base. Post-inflammatory hyperpigmentation is also observed. F) Pyoderma gangrenosum. The initial lesion is a non-follicular pustule, hemorrhagic, of rapid widening, surrounded by an erythematous strip. It’s very painful. Images are courtesy of the Dermatology Department of the University of Antioquia, Colombia. All images had the consent of the patients.

Table 3 summarizes the extra-intestinal cutaneous manifestations of IBD while Figure 1 shows examples of the different types of IBD.

**Table 3:** Summary of the extra-intestinal cutaneous manifestations of IBD Adapted from [10] IBD: inflammatory bowel disease

EIM	Epidemiology	Clinic	Pathophysiology	Disease course	Treatment
Erythema nodosum	CD>UC Up to 15% Female predominance	Subcutaneous nodules, elevated, sensitive, erythematous, or violaceous (1-5 cm); Extensor surface of lower limbs (anterior tibia)	Type IV hypersensitivity reaction; Trigger in 40%	Cicatrices self-limited; No scarring	Support measures: leg raise, analgesia, potassium iodide, compression socks, systemic corticosteroids if severe, anti-TNF in refractory cases
Pyoderma gangrenosum	UC>CD 0,4–2% Female predominance	Single or multiple erythematous papules/pustules, necrosis, or ulcers with purulent (sterile) discharge	Neutrophil function disorder, cellular immunity affected, pathergy	High recurrence rate, often weakening and severe (> 25%)	Local therapy (corticosteroids, tacrolimus, wet treatment), systemic corticosteroids, cyclosporin, anti-TNF, terapia local (corticosteroids, tacrolimus, wet treatment)
Sweet’s syndrome	Female predominance; IBD is the third most common disorder associated with Sweet's syndrome	Painful exanthema, papulosquamous, or nodules located in arms, legs, torso, hands, and face	Histocompatibility antigen association; Associated with different systemic diseases	Acute onset, associated with arthritis, fever, and ocular symptoms; No scarring	Topical or systemic corticosteroids Immunomodulators
Oral mucosa involvement	CD> UC up to 10% In 25% of cases, appears before IBD diagnosis	Aphthous stomatitis: painful and round/oval ulcers (mouth or lip mucosa) Periodontitis: gingival flushing, bleeding, edema. Peristomatitis vegetans: friable pustules with ulcers and hemorrhagic erosions.	Immune complex, aberrant immune response	Ulcers: no scarring if minor (<10 mm). Peristomatitis vegetans as the most severe and weakening form	IBD treatment, antiseptic mouth wash, and topical corticosteroids

## Conclusions

The prevalence and incidence of IBD are rising over the last few years. Early diagnosis and prompt treatment are fundamental to reduce morbidity and mortality in these patients. The presence of IBD-related skin lesions may prompt physicians to two thoughts: first, as cutaneous manifestations can occur before IBD symptoms in a non-negligible proportion, patients have to be screened for non-detected IBD even in the absence of symptoms; second, as most manifestations parallel intestinal disease activity, patients must be examined for intestinal disease activity even in the absence of symptoms. Therefore, it’s fundamental that all medical personnel are acquainted with cutaneous manifestations.
